# Supplementary material for: Parkour and Intrinsic Motivation: An Exploratory Multimethod Analysis of Self-Determination Theory in an Emerging Sport
Source: Int J Environ Res Public Health. 2025 Oct 27;22(11):1632. doi: 10.3390/ijerph22111632 (PMC12652841; doi:10.3390/ijerph22111632)
Supplement: Supplementary file 1 [file ijerph-22-01632-s001.zip › ijerph-3911220-supplementary.pdf]

File S1. Semi-Structured Interview Guide

- **Ice-breaker questions**: *The intent of this section is to encourage participants to talk freely about their personal experience with physical activity in a more open-ended, non-threatening manner.*
    - What are your favorite ways to be physically active?
        - Define Physical Activity as "The ways that you like to exercise or move around, this includes walking, biking, playing, etc."
        - How does being active make you feel?
        - Where do you practice Parkour or other activities?
            - If they do not practice outside, ask them why they feel it is hard to do that.

- **Autonomy and Perceptions**: *The intent of this section is to understand participants reasoning for participating in Parkour, and their perceptions of their autonomy in that choice and their other physical activity choices.*
    - How did you first get involved with Parkour?
        - If no name of a person is given, probe for how they first heard about it
    - What kinds of things make you excited to come to Parkour classes?
        - If they say that they are not excited, probe for why
        - What kinds of things make you not want to come to class?
        - If positive, ask what other ways they practice Parkour outside of classes.
    - Have you ever felt pressured or pushed by anyone to practice Parkour? Can you tell me more about that?
        - Maybe your parents, your friends, or even yourself?
    - What do friends or family think about you doing Parkour?
        - How do their opinions impact your physical activity choices?
    - What do you specifically like about Parkour compared to other activities?

- **Goals and Motivations**: *The intent of this section is to understand what the participants want to get out of Parkour, and how they view its role in their future.*
    - What do you want to get out of Parkour?
        - How long are you planning on doing Parkour?
    - What have you learned from Parkour that you can apply to life in general?
        - If they need priming; "How does Discipline play into this? Motivation? Teamwork?"

- **Competition**: *The intent of this section is to understand how participants relate to competition and how that impacts the physical activities they choose to engage in.*
    - How do you feel about competition in sports or activities other than Parkour?
        - Competing with friends? Competing with new people?
